# Supplementary material for: Rescue of mitochondrial import failure by intercellular organellar transfer
Source: Nat Commun. 2024 Feb 2;15:988. doi: 10.1038/s41467-024-45283-2 (PMC10837123; doi:10.1038/s41467-024-45283-2)
Supplement: Supplementary file 1 — Supplementary Information [file 41467_2024_45283_MOESM1_ESM.pdf]

Rescue of mitochondrial import failure by intercellular organellar transfer

Hope I. Needs[1], Emily Glover[1], Gonçalo C. Pereira[1,†], Alina Witt[2], Wolfgang Hübner[2], Mark P. Dodding[1], Jeremy M. Henley[1,*] and Ian Collinson[1,*]

[1] *School of Biochemistry, University of Bristol, Bristol BS8 1TD, UK*

[2] *Fakultät für Physik, Universität Bielefeld, Postfach 100131 D-33501, Germany*

[*], corresponding authors: J.M.Henley@bristol.ac.uk and ian.collinson@bristol.ac.uk

[†], present address: Nanna Therapeutics, Merrifield Centre, Rosemary Lane, Cambridge CB1 3LQ, UK.

This PDF file includes:

Supplementary Figures 1 to 12

Supplementary Table 1

Other Supplementary Materials for this manuscript include the following:

Supplementary Data

Supplementary Movie

Description of Additional Supplementary Information

Source Data

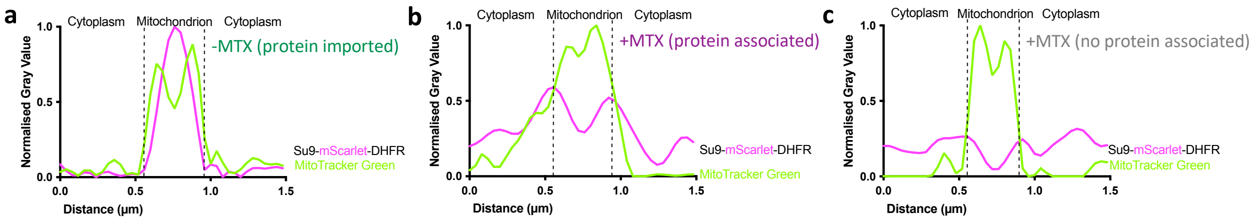

Supplementary Figure 1.

Representative plots of average pixel intensity across a mitochondrion in cells not treated with MTX (with imported precursor) **(a)** or subjected to the trapping insult (+MTX) with **(b)** or without **(c)** associated trapped precursor (48 h trapping). When the precursor is imported **(a)**, the mScarlet fluorescence is clearly within the mitochondrial matrix (between the MitoTracker Green peaks at the mitochondrial envelope), while in the presence of MTX when the precursor is associated with the mitochondrion **(b)**, there are two mScarlet peaks at the edge of the mitochondrion. When the protein is not associated with mitochondria in the presence of MTX **(c)**, there are no mitochondrial mScarlet peaks, as the signal is cytosolic. Source data are provided in the Source Data file. MTX, methotrexate.

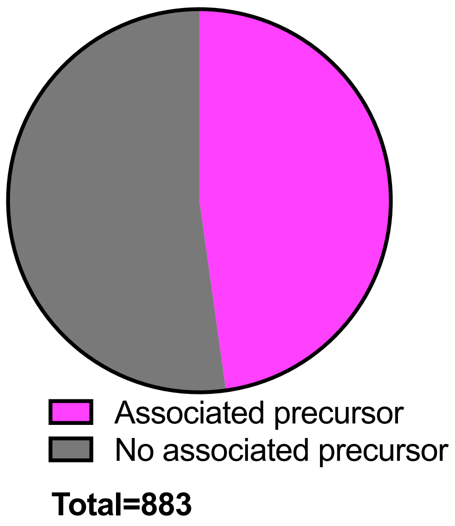

Supplementary Figure 2.

Quantification of the proportion of mitochondria per cell with (magenta) or without (grey) trapped precursor surrounding them. Analysis included a total of 883 mitochondria from N=3 biological replicates. Source data are provided in the Source Data file.

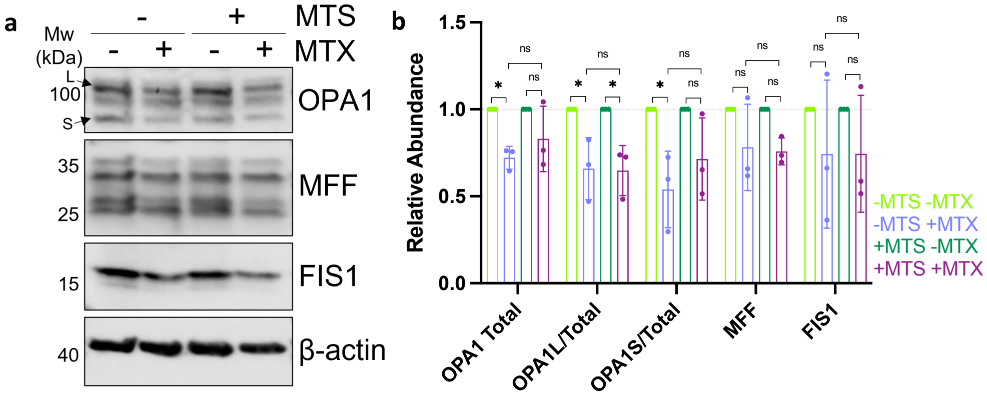

Supplementary Figure 3.

(a) Representative Western blots showing the abundance of total, long (L), and short (S) isoforms of OPA1, MFF, and FIS1 in HeLaGAL cells over-producing EGFP-DHFR or Su9-EGFP-DHFR (±MTS) in the absence or presence of 100 nM MTX for 48 h. β-actin was used as a loading control. N=5 biological replicates. Uncropped blots are provided in the Source Data file. **(b)** Quantification of (A). Data was normalised to β-actin loading control and to its respective ±MTX control, to account for the off-target effects of MTX treatment on cells. OPA1 L and S isoforms were quantified as isoform/total OPA1 to account for any changes in total OPA1 abundance. N=5 biological replicates. Statistical significance was determined using a one-way ANOVA with Tukey's test (B). Data are presented as mean values ± S.D. with individual data points for each biological replicate (B). Source data are provided in the Source Data file (B). kDa, kilodalton; MTS, mitochondrial targeting sequence; MTX, methotrexate; Mw, molecular weight.

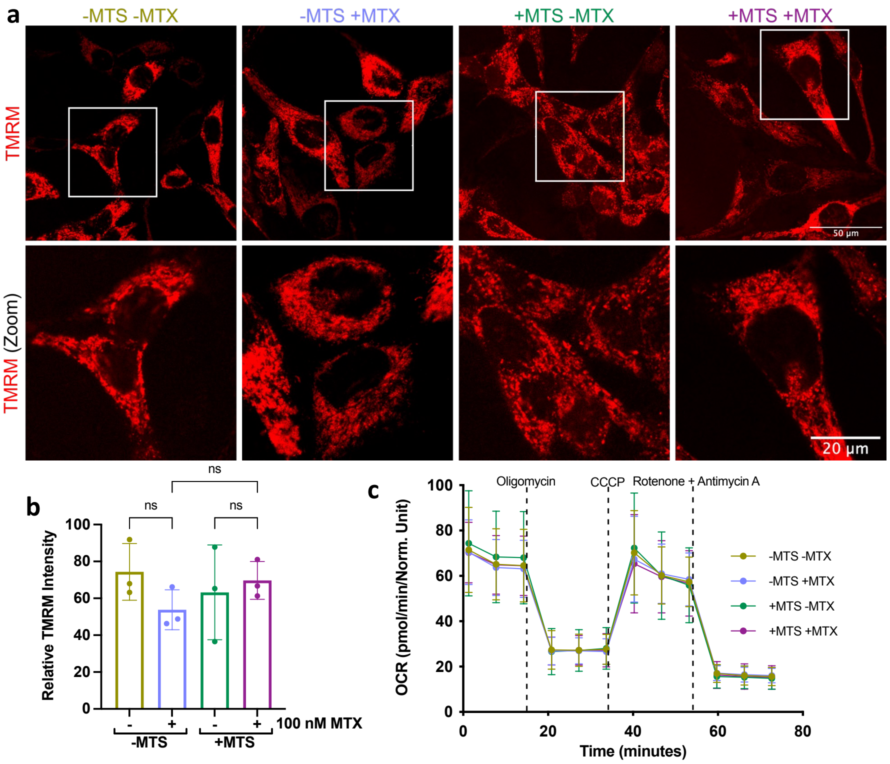

Supplementary Figure 4.

(a) Representative confocal images showing TMRM fluorescence (red) in the mitochondria of HeLaGAL cells over-producing EGFP-DHFR (-MTS) or Su9-EGFP-DHFR (+MTS) ±100 nM MTX (48 h). N=4 biological replicates. **(b)** Quantification of TMRM intensity in (**a**). **(c)** Mitochondrial stress test showing the OCR of HeLaGAL cells over-producing EGFP-DHFR (-MTS) or Su9-EGFP-DHFR (+MTS) ±100 nM MTX (48 h). N=6 biological replicates, each with 3 technical replicates. Statistical significance was determined using a one-way ANOVA with Tukey's test (**b**). Data are presented as mean values ± S.D. (**b**, **c**) with individual data points for each biological replicate (**b**). Source data are provided in the Source Data file (**b**, **c**). CCCP, carbonyl cyanide m-chlorophenyl hydrazone; MTS, mitochondrial targeting sequence; MTX, methotrexate; OCR, oxygen consumption rate; TMRM, tetramethylrhodamine methyl ester.

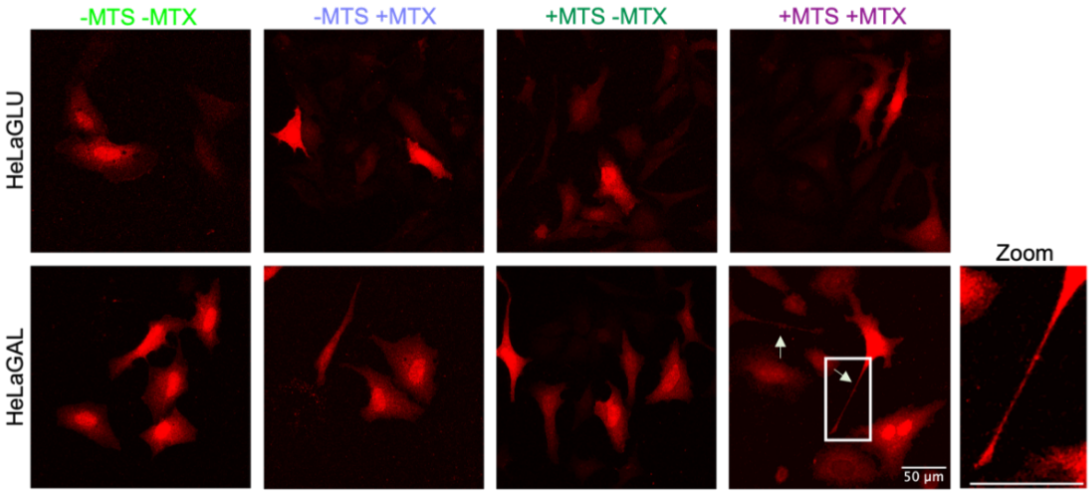

Supplementary Figure 5.

Representative confocal images showing the morphology (mCherry; red) of HeLaGLU or HeLaGAL cells over-producing EGFP-DHFR (-MTS) or Su9-EGFP-DHFR (+MTS) ±100 nM MTX (48 h). Arrows show protrusions and box shows zoom area. N=4 biological replicates. HeLaGAL, HeLa cells cultured in galactose-containing media; HeLaGLU, HeLa cells cultured in glucose-containing media; MTS, mitochondrial targeting sequence; MTX, methotrexate.

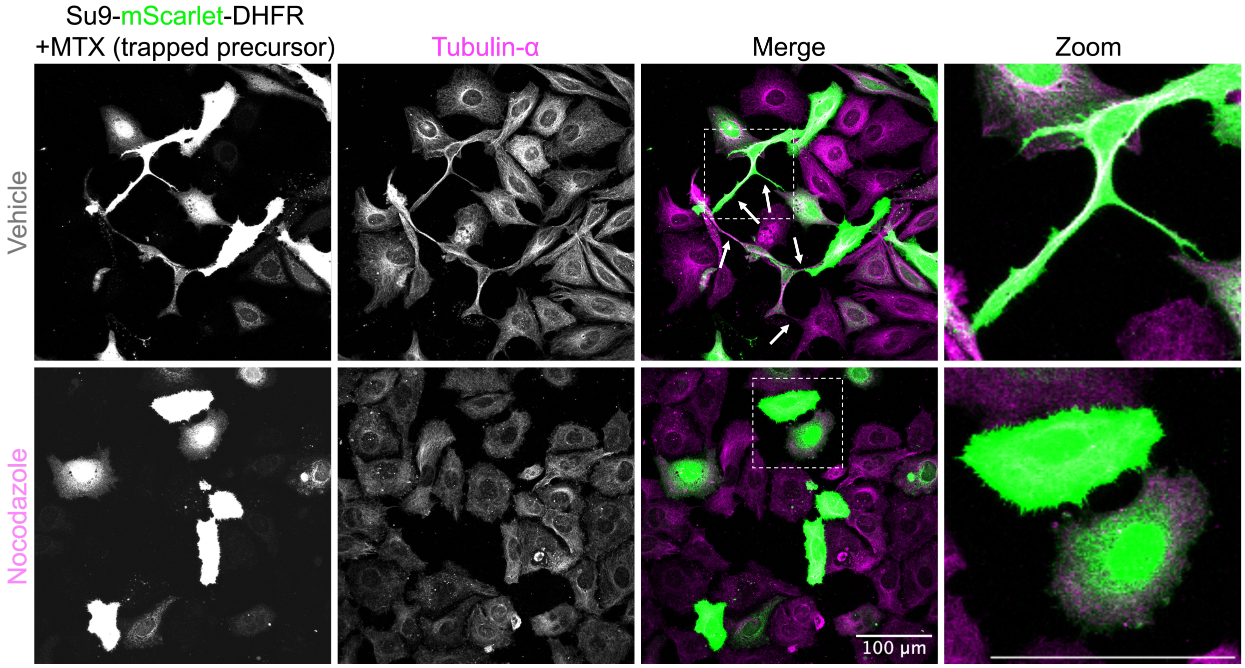

Supplementary Figure 6.

Representative confocal images of HeLaGAL cells exposed to precursor trapping (mScarlet, green; 48 h) ±100 nM nocodozole or vehicle (DMSO; 48 h) and stained for tubulin-α (magenta). Arrows show TNTs and box shows zoom area. N=3 biological replicates. MTX, methotrexate.

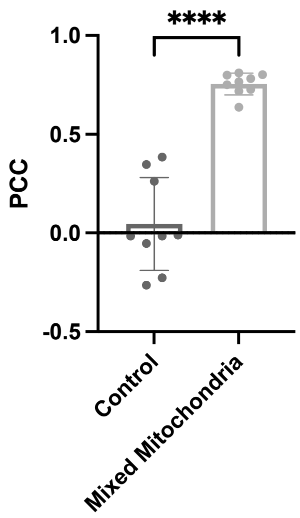

Supplementary Figure 7.
Quantification of the colocalisation of mitochondrial markers following mitochondrial transfer. PCC values were analysed (where 0 represents no correlation (no mixing) and 1 represents perfect positive correlation (mixing of mitochondrial markers)). N=3 biological replicates, n=3 cells analysed per biological replicate. Statistical significance was determined using a two-tailed, unpaired t-test. Data are presented as mean values ± S.D., and individual data points for each analysed cell are shown. Source data are provided in the Source Data file. PCC, Pearson correlation coefficient.

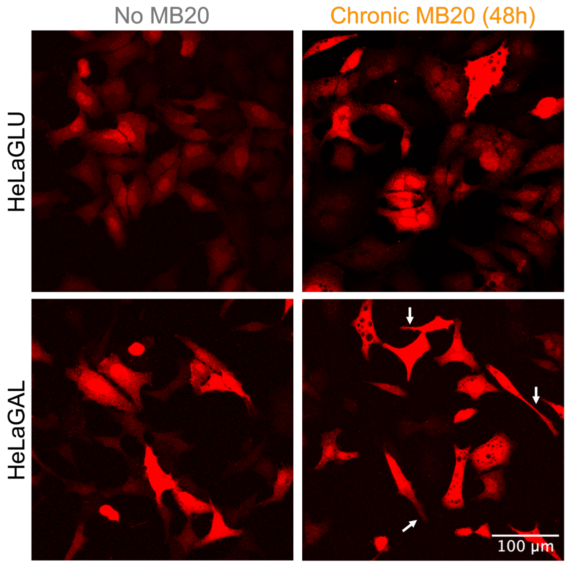

Supplementary Figure 8.

Representative confocal images showing HeLaGLU or HeLaGAL morphology (mCherry; red) following incubation with vehicle (DMSO) or 10 µM MB20 for 48 h. Arrows show TNTs. N=6 biological replicates. HeLaGAL, HeLa cells cultured in galactose-containing media; HeLaGLU, HeLa cells cultured in glucose-containing media; MB20, MitoBloCK-20.

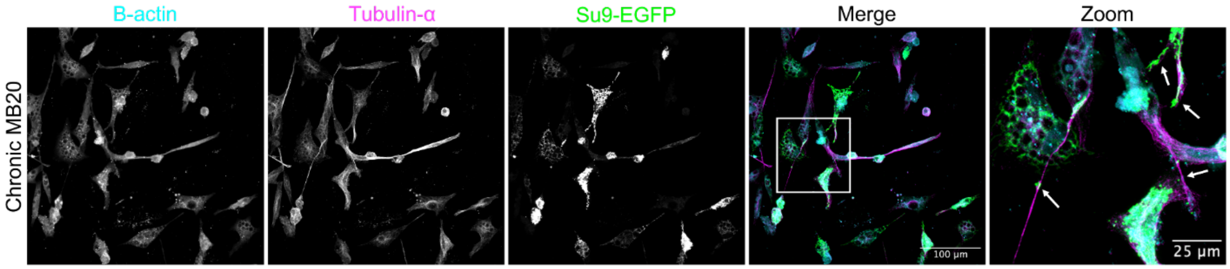

Supplementary Figure 9.

Representative confocal images showing β-actin (cyan) and tubulin-α (magenta) composition of MB20-induced protrusions. Box shows zoom area, and arrows show TNTs. N=3 biological replicates. MB20, MitoBloCK-20.

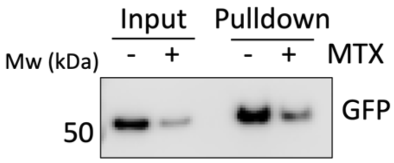

Supplementary Figure 10.

Representative Western blot showing GFP-tagged protein in the input and pulldown samples. N=3 biological replicates. The uncropped blot is provided in the Source Data file. kDa, kilodalton; MTX, methotrexate; Mw, molecular weight.

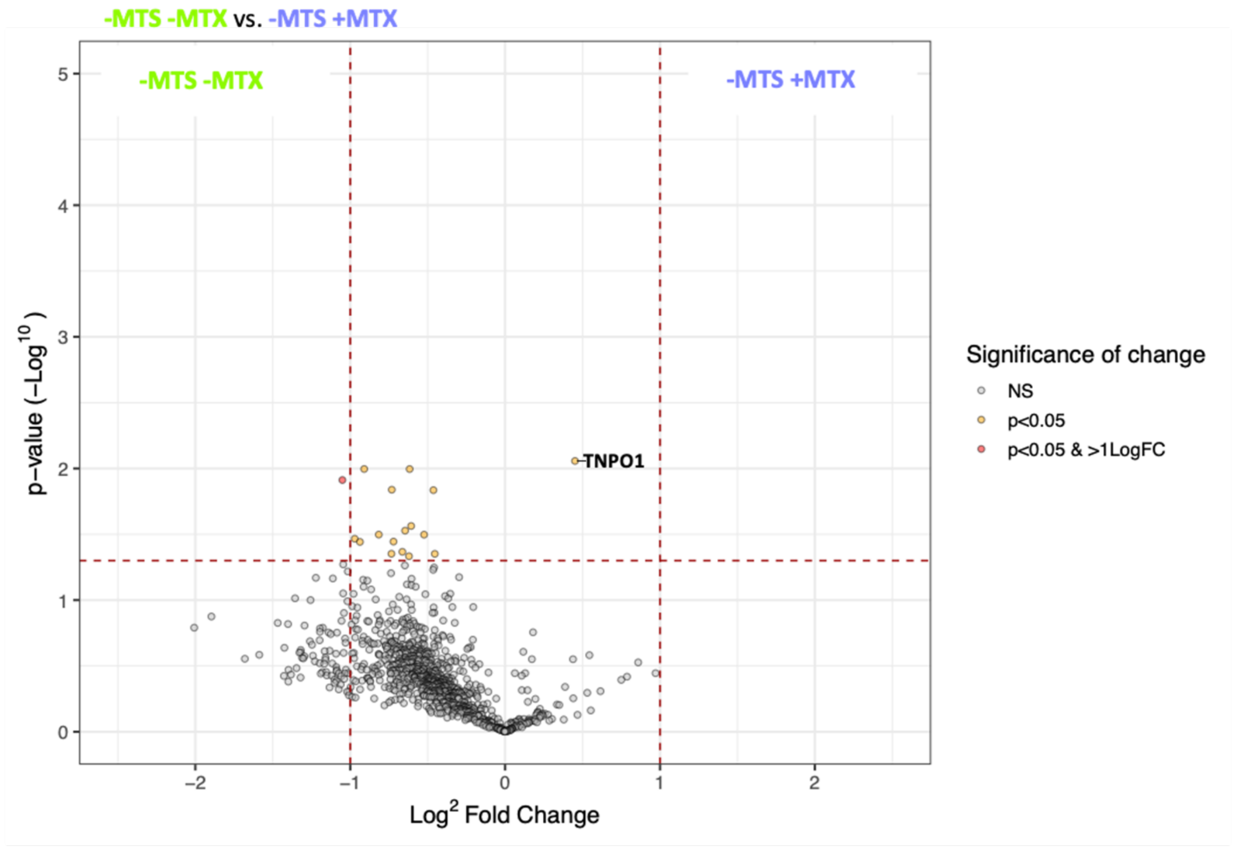

Supplementary Figure 11.

Volcano plot highlighting proteins enhanced in pulldown samples from mitochondria of cells over-producing EGFP-DHFR (-MTS) ± 100 nM MTX (48 h). The single protein enhanced in the presence of MTX is highlighted with its gene name (TNPO1) on the volcano plot. N=3 biological replicates. Statistical significance was determined using a two-tailed, unpaired t-test. Source data is provided in the Supplementary Data file. LogFC, Log^2 fold change; MTS, mitochondrial targeting sequence; MTX, methotrexate; NS, not significant.

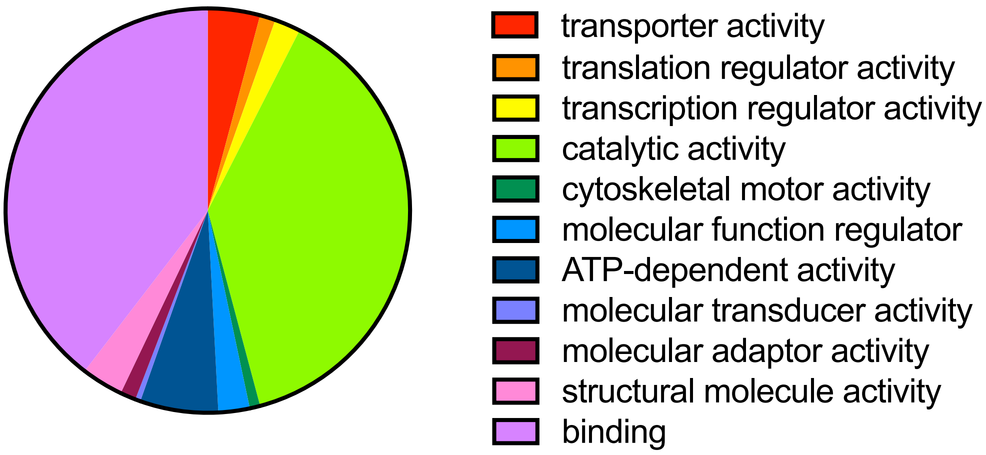

Supplementary Figure 12.
Representation of the possible cellular pathways associated with precursor trapping, identified using the PANTHER classification system. Source data is provided in the Supplementary Data file.

Supplementary Table 1.

Tabular representation of the possible cellular pathways associated with precursor trapping, identified using the PANTHER classification system. Mitochondrial proteins were identified using the MitoCoP database as a reference. Source data is provided in the Supplementary Data file.

Function	Number of Proteins	Proportion of Total (%)
Transporter Activity	10	4.2
Translation Regulator Activity	3	1.3
Transcription Regulator Activity	5	2.1
Catalytic Activity	92	38.3
Cytoskeletal Motor Activity	2	0.8
Molecular Function Regulator	6	2.5
ATP-Dependent Activity	15	6.3
Molecular Transducer Activity	1	0.4
Molecular Adaptor Activity	3	1.3
Structural Molecule Activity	8	3.3
Binding	95	39.6
Mitochondrial Proteins	55	21.3
