# Supplementary material for: Rescue of mitochondrial import failure by intercellular organellar transfer
Source: Nat Commun. 2024 Feb 2;15:988. doi: 10.1038/s41467-024-45283-2 (PMC10837123; doi:10.1038/s41467-024-45283-2)
Supplement: Supplementary file 3 — Description of Additional Supplementary Files [file 41467_2024_45283_MOESM3_ESM.docx]

Supplementary Movie.

Time-lapse movie showing the transfer of healthy mitochondria (MitoTracker Green; green) within a TNT into a cell with trapped precursor (Su9-mScarlet-DHFR (magenta) +100 nM MTX; 48 h treatment). HeLaGAL cells were imaged live using an Olympus IXplore SpinSR system. Each timeframe is a Max z-projection of 10 slices, taken every 5 minutes with 10 time points.

Supplementary Data.

Analysis of TMT-MS data, showing all proteins associated with the pulled-down proteins (Su9-EGFP-DHFR or EGFP-DHFR +/-MTX) in the mitochondrial fractions of HeLaGAL cells (sheet 1). MitoCoP analysis (sheet 2). Protein sequences (sheet 3).
