# Supplementary material for: Rescue of mitochondrial import failure by intercellular organellar transfer
Source: Nat Commun. 2024 Feb 2;15:988. doi: 10.1038/s41467-024-45283-2 (PMC10837123; doi:10.1038/s41467-024-45283-2)
Supplement: Supplementary file 6 — Reporting Summary [file 41467_2024_45283_MOESM6_ESM.pdf]

nature portfolio

Corresponding author(s): Ian Collinson

Last updated by author(s): 26/07/23 XXXXXX 18/12/23

Reporting Summary

Nature Portfolio wishes to improve the reproducibility of the work that we publish. This form provides structure for consistency and transparency in reporting. For further information on Nature Portfolio policies, see our Editorial Policies and the Editorial Policy Checklist.

Statistics

For all statistical analyses, confirm that the following items are present in the figure legend, table legend, main text, or Methods section.

n/a	Confirmed	
☐	☒	The exact sample size (*n*) for each experimental group/condition, given as a discrete number and unit of measurement
☐	☒	A statement on whether measurements were taken from distinct samples or whether the same sample was measured repeatedly
☐	☒	The statistical test(s) used AND whether they are one- or two-sided<br>*Only common tests should be described solely by name; describe more complex techniques in the Methods section.*
☒	☐	A description of all covariates tested
☐	☒	A description of any assumptions or corrections, such as tests of normality and adjustment for multiple comparisons
☐	☒	A full description of the statistical parameters including central tendency (e.g. means) or other basic estimates (e.g. regression coefficient) AND variation (e.g. standard deviation) or associated estimates of uncertainty (e.g. confidence intervals)
☒	☐	For null hypothesis testing, the test statistic (e.g. *F*, *t*, *r*) with confidence intervals, effect sizes, degrees of freedom and *P* value noted<br>*Give P values as exact values whenever suitable.*
☒	☐	For Bayesian analysis, information on the choice of priors and Markov chain Monte Carlo settings
☒	☐	For hierarchical and complex designs, identification of the appropriate level for tests and full reporting of outcomes
☒	☐	Estimates of effect sizes (e.g. Cohen's *d*, Pearson's *r*), indicating how they were calculated

Our web collection on statistics for biologists contains articles on many of the points above.

Software and code

Policy information about availability of computer code

Data collection	No code was used for data collection.
Data analysis	Code was used for some data analysis (mitochondrial pre-processing and network analysis). This is referenced in the Methods wherever applicable with links to the GitHub or Zenodo pages for access to the code. A description of how the analysis worked is also provided in the methods section.

For manuscripts utilizing custom algorithms or software that are central to the research but not yet described in published literature, software must be made available to editors and reviewers. We strongly encourage code deposition in a community repository (e.g. GitHub). See the Nature Portfolio guidelines for submitting code & software for further information.

Data

Policy information about availability of data

All manuscripts must include a data availability statement. This statement should provide the following information, where applicable:

- Accession codes, unique identifiers, or web links for publicly available datasets
- A description of any restrictions on data availability
- For clinical datasets or third party data, please ensure that the statement adheres to our policy

All data are available in the main text or the supplementary materials.
Supplementary Materials contains Supplementary Figures 1-18, Supplementary Table 1, and caption for Supplementary Movie 1.

Supplementary Movie 1 is included as a separate downloadable file.
Proteomics data are available via ProteomeXchange with identifier PXD040098.
All source data, including uncropped Western blots, are available in the Source Data file.

Human research participants

Policy information about studies involving human research participants and Sex and Gender in Research.

Reporting on sex and gender	No research was conducted on human participants.
Population characteristics	No research was conducted on human participants.
Recruitment	No research was conducted on human participants.
Ethics oversight	No research was conducted on human participants.

Note that full information on the approval of the study protocol must also be provided in the manuscript.

Field-specific reporting

Please select the one below that is the best fit for your research. If you are not sure, read the appropriate sections before making your selection.

☒ Life sciences ☐ Behavioural & social sciences ☐ Ecological, evolutionary & environmental sciences

For a reference copy of the document with all sections, see nature.com/documents/nr-reporting-summary-flat.pdf

Life sciences study design

All studies must disclose on these points even when the disclosure is negative.

Sample size	No sample size calculations were performed. Sample sizes chosen in accordance with experience and laboratory standard operating procedures, sufficient for statistical analysis and significance / ns determination. Unnecessary repeats were avoided in the interests of time.
Data exclusions	No data was excluded.
Replication	All experiments had at least 3 biological replicates, and where applicable (for example when running plate reader assays), 3 technical replicates were also included for each biological replicate (i.e. 3 wells were measured for each condition). N represents a biological replicate, while n represents i.e. number of cells/mitochondria where multiple were counted per biological replicate. All attempts of replication were successful.
Randomization	Not relevant to this study; all experiments were carried out in cell lines.
Blinding	Where image analysis was done manually (i.e. quantifying TNTs from confocal images), images were blinded prior to analysis, to avoid any bias in data analysis.

Reporting for specific materials, systems and methods

We require information from authors about some types of materials, experimental systems and methods used in many studies. Here, indicate whether each material, system or method listed is relevant to your study. If you are not sure if a list item applies to your research, read the appropriate section before selecting a response.

Materials & experimental systems

n/a	Involved in the study	
☐	☒	Antibodies
☐	☒	Eukaryotic cell lines
☒	☐	Palaeontology and archaeology
☒	☐	Animals and other organisms
☒	☐	Clinical data
☒	☐	Dual use research of concern

Methods

n/a	Involved in the study	
☒	☐	ChIP-seq
☒	☐	Flow cytometry
☒	☐	MRI-based neuroimaging

Antibodies

Antibodies used	β-actin (Sigma; A2228)

Antibodies used	Tubulin-α (BioRad; MCA78G)<br>GFP (Sigma; G1544)<br>VDAC (Invitrogen; PA1-954A)<br>RhoGDI (Abcam; ab133248)<br>DRP1 (BD Biosciences; 6111113)<br>phospho-DRP1 S616 (CST; 4494)<br>OPA1 (Abcam; ab42364)<br>MFF (Proteintech; 17090-1-AP)<br>FIS1 (Proteintech; 10956-1-AP)
Validation	All antibodies had been previously validated for the required application, and were used in line with references on the suppliers' website.

Eukaryotic cell lines

Policy information about cell lines and Sex and Gender in Research

Cell line source(s)	HeLa cells - ATCC; HEK293T - ECACC
Authentication	Cell lines were not authenticated but were bought from reputable sources - ATCC and ECACC.
Mycoplasma contamination	All cell lines were regularly tested for mycoplasma using Eurofins mycoplasma testing service.
Commonly misidentified lines<br>(See ICLAC register)	No commonly misidentified cell lines were used in this study.
